# Splenic metastasis from small bowel adenocarcinoma

**DOI:** 10.1093/jscr/rjaf1001

**Published:** 2025-12-18

**Authors:** Adelina E Coturel, Paula R Pereyra, Rodrigo P Garcia, Agustin Diomedi, Alejandro Lopez Presa

**Affiliations:** Department of Surgery, Hospital del Bicentenario de Esteban Echeverría, Esteban Echeverria 1842, Buenos Aires, Argentina; Department of Surgery, Hospital del Bicentenario de Esteban Echeverría, Esteban Echeverria 1842, Buenos Aires, Argentina; Department of Surgery, Hospital del Bicentenario de Esteban Echeverría, Esteban Echeverria 1842, Buenos Aires, Argentina; Department of Oncology, Hospital del Bicentenario de Esteban Echeverría, Esteban Echeverria 1842, Buenos Aires, Argentina; Department of Pathology, Hospital del Bicentenario de Esteban Echeverría, Esteban Echeverria 1842, Buenos Aires, Argentina

**Keywords:** small bowel adenocarcinoma, splenic metastasis, laparoscopic splenectomy, PET/CT, case report

## Abstract

Small bowel adenocarcinoma is a rare malignancy, often diagnosed at advanced stages due to nonspecific symptoms. Common metastatic sites include the liver and peritoneum, while splenic involvement has not been previously documented. We report the case of a 42-year-old woman with chronic anemia and dyspepsia, who later presented with overt gastrointestinal bleeding. Imaging revealed a stenotic jejunal lesion, hypermetabolic mesenteric lymphadenopathy, and a hypermetabolic splenic nodule on positron emission tomography–computed tomography (PET/CT). She underwent laparoscopic segmental jejunal resection with lymphadenectomy, followed by laparoscopic splenectomy. Histopathology confirmed a moderately differentiated adenocarcinoma with nodal involvement (pT3 pN2) and an enteric-type adenocarcinoma infiltrating the spleen. The patient recovered uneventfully and is scheduled for adjuvant chemotherapy. This case highlights the diagnostic challenges of small bowel adenocarcinoma, the role of PET/CT in staging, and the feasibility of laparoscopic splenectomy for rare isolated splenic metastases. Long-term follow-up will determine its prognostic impact.

## Introduction

Small bowel tumors represent 3%–6% of gastrointestinal neoplasms, with prevalence likely increasing due to improved diagnostic modalities [1]. Small bowel adenocarcinoma (SBA) and carcinoid tumors are the most common subtypes, while lymphoma should be considered in patients with celiac disease and enlarged mesenteric nodes. SBA most often arises in the duodenum or jejunum, whereas lymphoma is more frequent in the ileum [2].

Among SBA patients, ~32% present with stage IV disease at diagnosis. The liver and peritoneum are the most common metastatic sites, following patterns seen in other gastrointestinal cancers. Although isolated brain [3] and ovarian [4] metastases have been reported, splenic metastasis from SBA has not, to our knowledge, been documented.

Given the diagnostic challenges of SBA and the rarity of splenic involvement, this case adds a novel presentation to the literature and may broaden understanding of its metastatic patterns. Management of metastatic SBA remains controversial, particularly regarding the role of metastasectomy in isolated or oligometastatic disease, due to limited evidence and poor prognosis in advanced stages [5].

In this report, we describe a unique case of SBA with solitary splenic metastasis, an exceptionally rare metastatic presentation not yet reported in the literature.

## Case report

A 42-year-old female patient had been under evaluation for 2 years due to chronic anemia and dyspepsia. Initial diagnostic workup, including upper and lower endoscopies and a contrast-enhanced CT scan, showed no pathological findings.

She was recently admitted with an episode of overt gastrointestinal bleeding, manifested as melena, which required blood transfusion. A new contrast-enhanced CT scan revealed segmental wall thickening of the proximal jejunum, a 19-mm retroperitoneal lymph node, and a small amount of free fluid in the pouch of Douglas.

**Figure 1:**
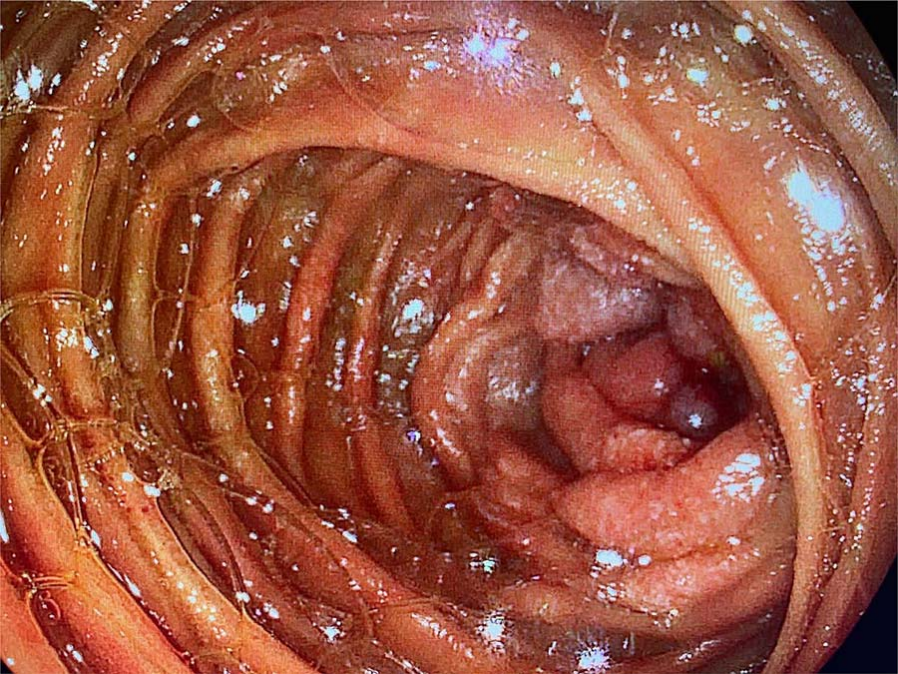
Upper endoscopy showing a proliferative, stenotic lesion in the jejunum.

An upper endoscopy using a colonoscope was performed to explore the proximal jejunal loops. A stenotic, proliferative lesion was identified in the first jejunal segments (Fig. 1).

**Figure 2:**
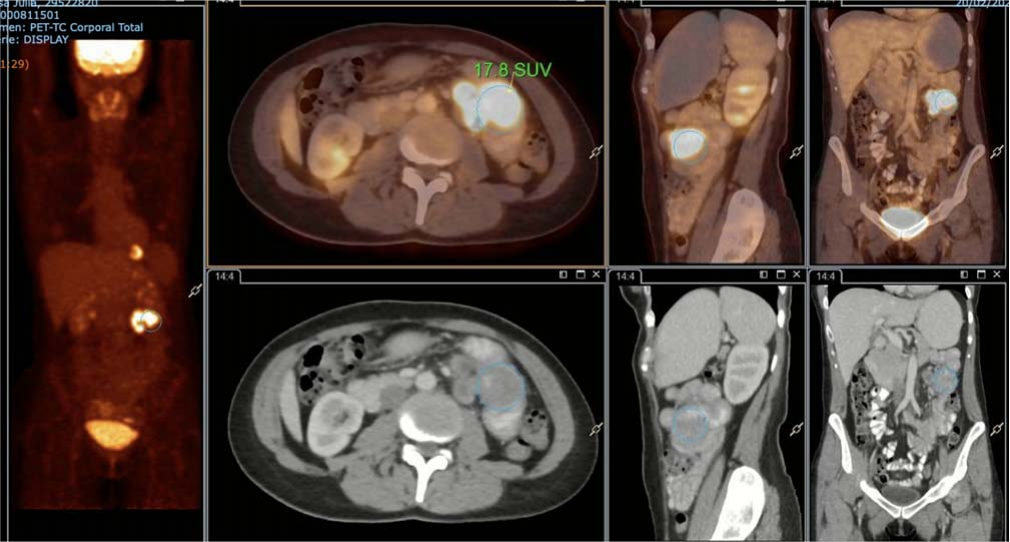
PET/CT showing jejunal wall thickening with increased uptake (SUVmax 17.8).

**Figure 3:**
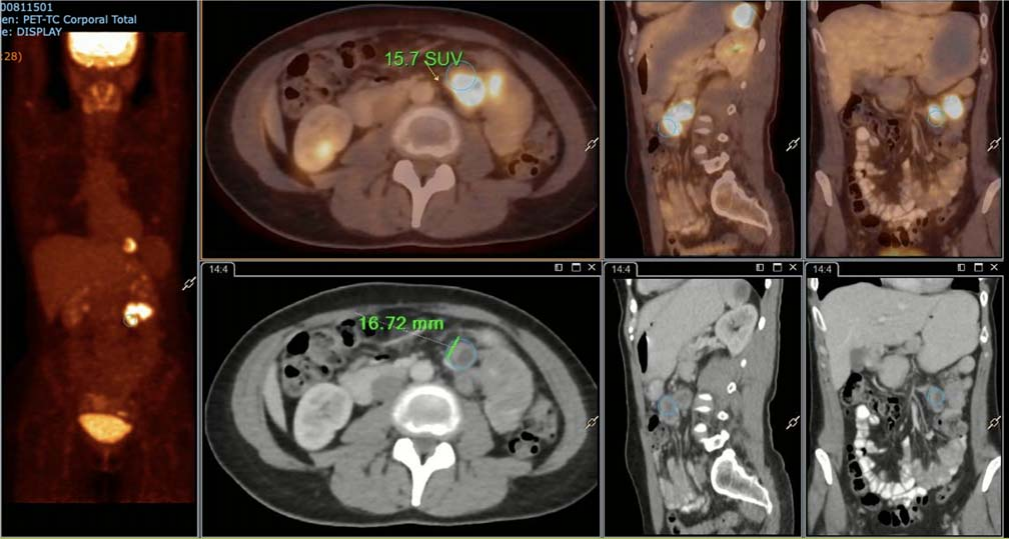
PET/CT showing a hypermetabolic peritumoral lymph node (SUVmax 15.7).

**Figure 4:**
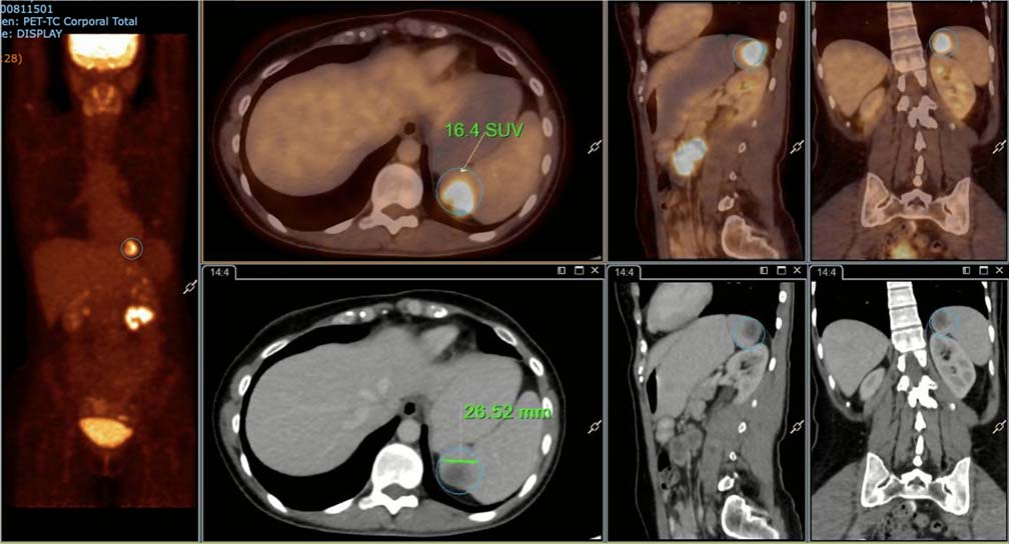
PET/CT showing a hypermetabolic splenic nodule in the upper pole (SUVmax 16.4).

A subsequent PET/CT, performed prior to any surgical intervention, demonstrated intense radiotracer uptake in the thickened jejunal wall (SUV 17.8), in hypermetabolic mesenteric lymph nodes adjacent to the lesion (SUV 15.7), and in a 26.5-mm hypermetabolic nodule in the upper pole of the spleen (SUV 16.4) (Figs 2–4). Given these findings, lymphoma was included in the preoperative differential diagnosis.

**Figure 5:**
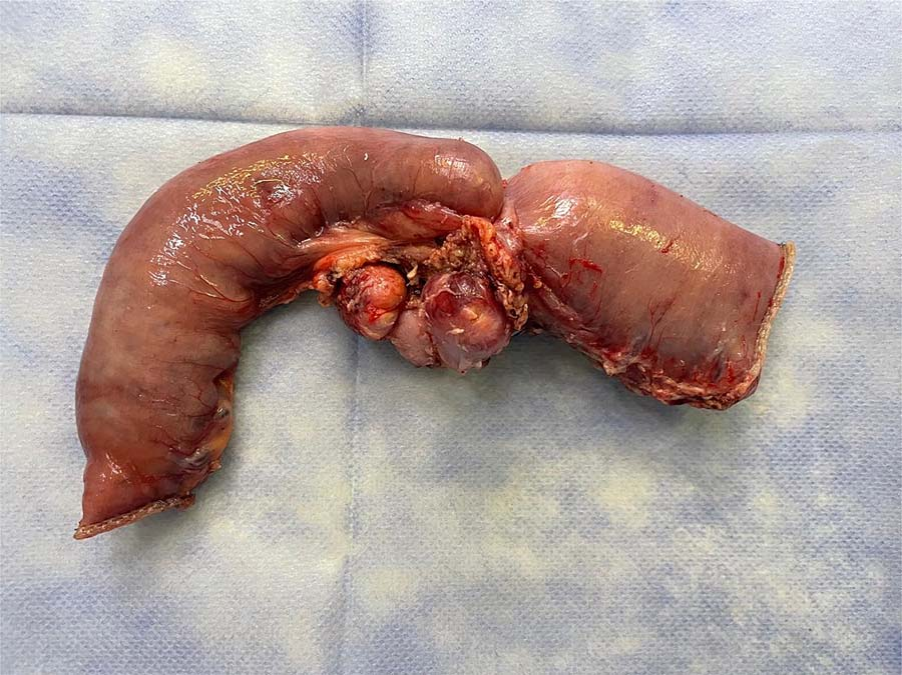
Surgical specimen showing a stenotic jejunal lesion with mesenteric lymph nodes.

Therefore, we proceeded with an initial surgical intervention to resect the affected bowel segment and obtain a definitive histopathological diagnosis. A laparoscopic approach revealed a stenotic lesion located 50 cm distal to the ligament of Treitz, associated with mesenteric lymphadenopathy. The affected segment and lymph nodes were resected en bloc, followed by an intracorporeal stapled anastomosis. The specimen (Fig. 5) was extracted through a Pfannenstiel incision.

Histopathological examination revealed a moderately differentiated (Grade 2) adenocarcinoma of the small intestine, infiltrating through the muscularis propria into the subserosal adipose tissue, with metastatic involvement of three lymph nodes (pT3 pN2). Immunohistochemical analysis showed no evidence of microsatellite instability, indicating proficient mismatch repair (pMMR).

**Figure 6:**
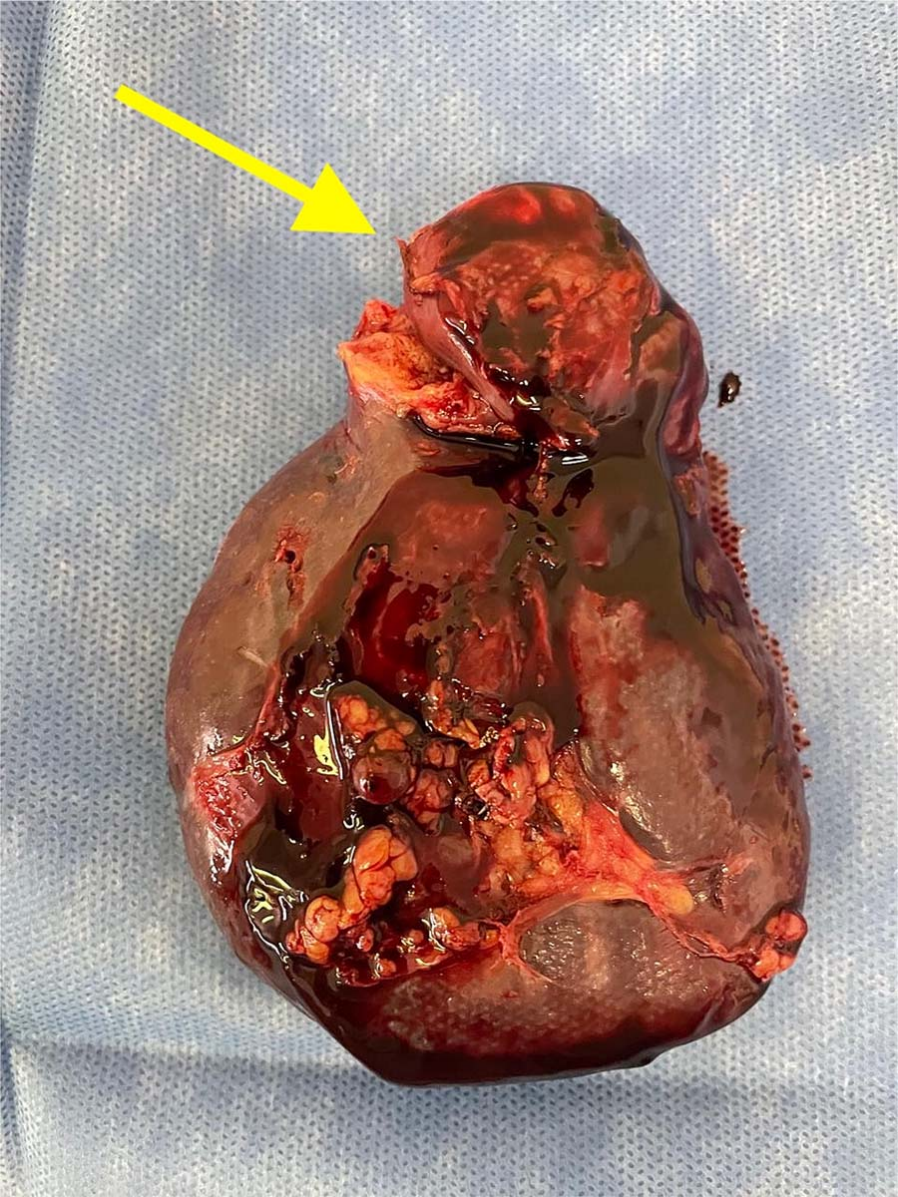
Splenectomy specimen showing the metastatic lesion in the upper pole (arrow).

**Figure 7:**
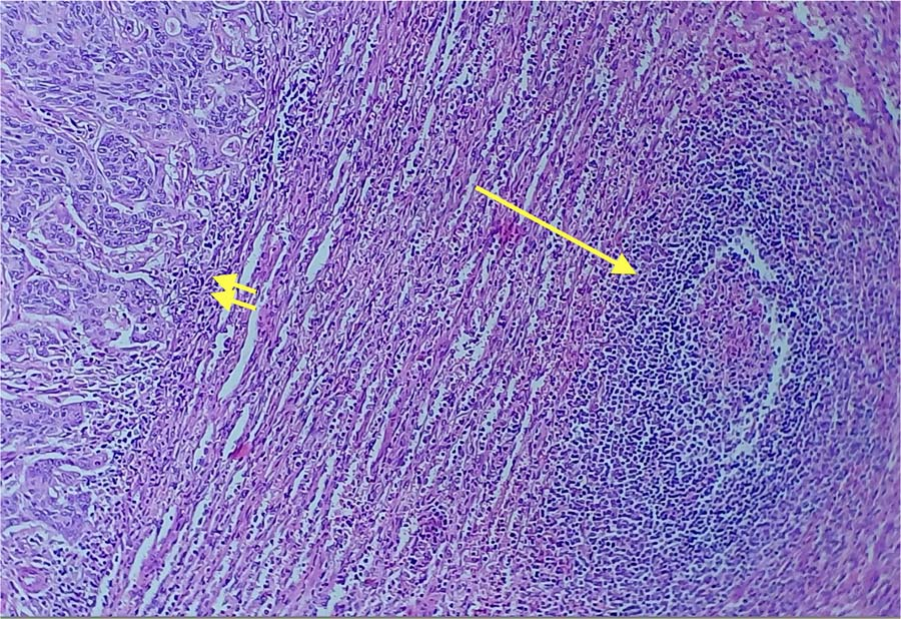
Red and white pulp of the splenic parenchyma (arrow) with infiltration by intestinal-type adenocarcinoma (double arrow, left); H&E ×200.

**Figure 8:**
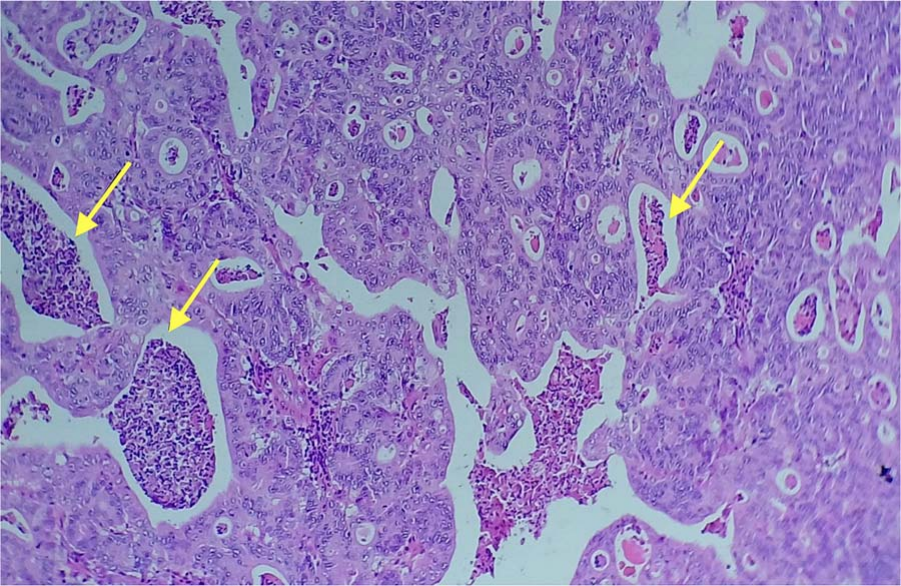
Area of enteric-type adenocarcinoma composed of neoplastic proliferation of columnar epithelial cells with nuclear atypia, eosinophilic cytoplasm, and a complex tubular and cribriform arrangement with endoluminal necrosis (arrows); H&E ×200.

**Figure 9:**
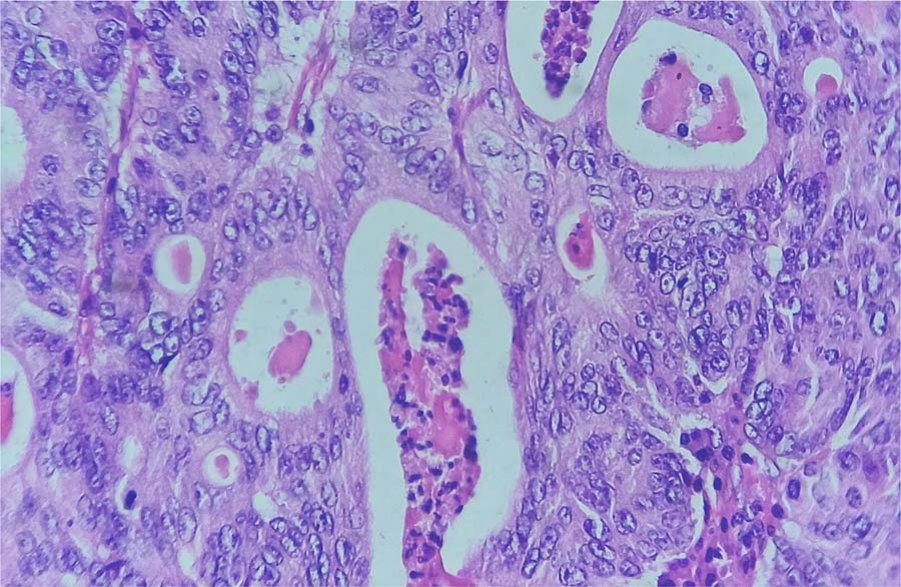
Detail of an area of enteric-type adenocarcinoma composed of neoplastic proliferation of columnar epithelial cells with nuclear atypia, eosinophilic cytoplasm, and a complex tubular and cribriform arrangement with endoluminal necrosis; H&E ×400.

Given the histopathological findings, a laparoscopic splenectomy was performed in a second operative session. The procedure was carried out in the left lateral decubitus position using three trocars. Intraoperatively, a 3 × 3 cm lesion was identified within the splenic parenchyma. The specimen (Fig. 6) was extracted through the previously used Pfannenstiel incision. Histopathological analysis confirmed infiltration by an enteric-type adenocarcinoma (Figs 7–9).

The patient recovered uneventfully from both procedures and has completed four cycles of adjuvant capecitabine and oxaliplatin (CAPOX) chemotherapy to date.

**Figure 10:**
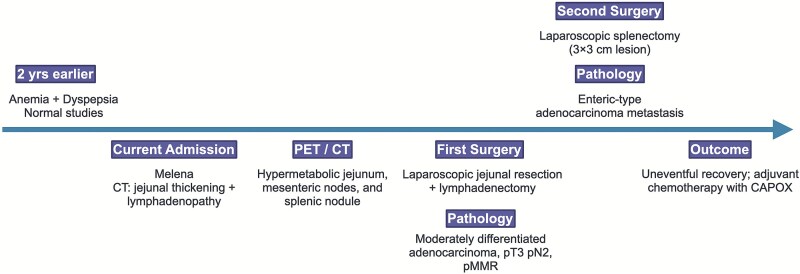
Timeline summarizing the patient’s clinical presentation, diagnostic workup, surgical management, and outcome.

Figure 10 provides a timeline summarizing the key events of the case.

## Discussion

We present the case of a patient with SBA and splenic metastasis. The diagnosis was challenging, as initial imaging and endoscopic studies yielded negative results. Although PET/CT is not routinely indicated in the initial workup of small bowel tumors, it may be useful for evaluating metastatic disease or when CT or MRI findings are inconclusive [2]. In our case, PET/CT played a key role by revealing a hypermetabolic splenic nodule, which was not clearly characterized on prior imaging studies. Furthermore, the unusual presentation of splenic involvement initially raised suspicion of lymphoma rather than adenocarcinoma.

Patients with metastatic disease have a poor prognosis, with a reported median overall survival (OS) of less than 20 months among those receiving chemotherapy [6]. To date, there are no randomized clinical trials evaluating the role of metastasectomy in this disease.

Bhamidipati *et al*. [7] reported the largest retrospective series of metastatic SBA. Of 437 patients, 77% had single-organ and 23% multiple-site metastases. The liver and peritoneum were most common, followed by lungs and distant lymph nodes. Seventy-five patients underwent metastasectomy, with a median OS of 34.5 months from diagnosis. Resection sites included peritoneum (65%), liver (21%), distant nodes (9%), and lungs (0.4%). On multivariate analysis, celiac disease, chemotherapy, metastasectomy, and deficient mismatch repair (dMMR) status correlated with improved survival. The authors concluded metastasectomy may benefit well-selected patients.

The ARCAD-NADEGE cohort [5] evaluated outcomes after SBA metastasectomy in 34 patients, 85% with a single metastatic site. Common locations were peritoneum, liver, lymph nodes, and lungs. Procedures included peritoneal metastasectomy ±  hyperthermic intraperitoneal chemotherapy (HIPEC), liver resection, radiofrequency ablation, lymph node dissection, and pulmonary lobectomy. Median OS was 28.6 months, with 41.2% surviving >36 months. Poor differentiation, positive margins, and lymph node involvement at primary surgery predicted worse survival. The authors concluded metastasectomy may be considered in patients with resectable disease, good performance status, and well-differentiated tumors.

Based on these findings, the 2025 NCCN Guidelines for Small Bowel Adenocarcinoma [8] suggest that select patients with limited visceral metastases may be candidates for metastasectomy, and that such cases should be evaluated by a multidisciplinary team.

Although no previous reports describe splenic metastasis from SBA, laparoscopic splenectomy has been shown to be a safe and feasible procedure for both benign and malignant splenic lesions [9]. In our case, the patient experienced no postoperative complications and benefited from a faster recovery due to the minimally invasive approach.

In conclusion, laparoscopic splenectomy may represent a safe and effective therapeutic option for isolated splenic metastases of small bowel adenocarcinoma. Long-term follow-up will help determine whether this intervention has a meaningful impact on OS.
